# Enhancing athlete performance under pressure: the role of attribution training in mitigating choking

**DOI:** 10.3389/fpsyg.2025.1435374

**Published:** 2025-02-07

**Authors:** Dan Huang, Huilin Wang, Yiwei Tang, Hanyue Lei, Denise Koh

**Affiliations:** ^1^Faculty of Education, Universiti Kebangsaan Malaysia, Bangi, Selangor, Malaysia; ^2^School of Physical Education, Hunan University of Science and Technology, Xiangtan, China; ^3^School of Business, Hunan University of Science and Technology, Xiangtan, China

**Keywords:** choking, attribution training, self-efficacy, fear of failure, self-criticism

## Abstract

**Introduction:**

Choking in athletes describes a marked reduction in their skill level, falling below the normal level, when under stress. This paradoxical performance decline, which athletes strenuously to avoid yet frequently encounter, was the focus of this study. We implemented training interventions on athletes who had experienced choking to assess their impact on fear of failure and self-criticism. Correct or improve the subjects’ inappropriate attribution of failure results, and enable them to learn to use appropriate and positive attribution, enhance motivation levels, and thus achieve the purpose of improving behavior and performance levels.

**Methods:**

The snowball sampling technique was used to collect data through the combination of online electronic questionnaires and offline paper questionnaires, so as to explore the choking phenomenon of elite athletes by attributing the influence of training variables on self-efficacy. The research team conducted a survey of elite athletes in Central China between October and December 2023. In this study, 350 questionnaires were distributed, 350 questionnaires were collected after the questionnaires were distributed, and 328 valid questionnaires were finally eliminated through screening. And the relevant statistical analysis is carried out on the data.

**Results:**

The results confirmed the significant correlations between attribution training and fear of failure (*β* = −0.548, *p* < 0.001), attribution training and self-criticism (*β* = −0.531, *p* < 0.001), fear of failure and self-efficacy (*β* = −0.240, *p* < 0.001), and self-criticism and self-efficacy (*β* = −0.408, *p* < 0.001). Finally, the correlation between fear of failure and self-criticism (*β* = −0.211, *p* < 0.01) was validated.

**Conclusion:**

Athletes who underwent attribution training showed significant reductions in fear of failure and self-criticism, thereby decreasing their negative emotions, enhancing their positive emotions, and improving their self-efficacy during sports performance.

## 1 Introduction

The pursuit of excellence in competitive sports is a constant goal for both athletes and coaches, aiming to perform at their best during competitions (Lynch, 2021). The rising pressure on athletes during their training sessions and contests is becoming increasingly evident (Low et al., 2023). Research has shown that the stress associated with competition can lead to a range of detrimental effects for both athletes individually and for sports organizations as a whole. These include anxiety, aggression, and a decrease in satisfaction, all of which can adversely affect their performance in competitions and their well-being (Brumels and Beach, 2008). Under competition stress, the phenomenon of choking during competition is a common polarized performance occurrence among athletes (Hsu et al., 2019). Choking refers to the deterioration of habitual motor execution, typically occurring at critical moments or in major competitions, and is characterized by uncharacteristic mistakes made by athletes under increased pressure (Hill et al., 2019). Athletes who have experienced choking often lose confidence in themselves, develop a fear of competition, negatively affect their team, and cause losses to their country’s overall competition results (Jordet, 2009). Considering the distinct challenges and pressures faced by athletes in today’s competitive sports landscape, exploring strategies to prevent performance declines under pressure has become a significant focus in sports psychology research (Low et al., 2023).

In competitive sports, most athletes have experienced the phenomenon of choking, which runs counter to the ideal state of competition (Mesagno and Beckmann, 2017). Stress is not only interpreted as a direct cause of performance decline among athletes but also serves as an important marker distinguishing other forms of sports performance (Belletier et al., 2015). Since choking emphasizes the abnormal performance of athletes under stress, describing errors that should not occur due to psychological changes, and considering that such stress-related errors are not only prevalent in sports competitions but also in exams, performances, firefighting, policing, combat, and emergency rescue situations (Nieuwenhuys and Oudejans, 2011), the exploration of the choking phenomenon has attracted significant attention from psychologists and researchers.

A review of past studies indicates that much of the research on helping athletes who have experienced choking to return to competitive sports, has focused on external factors, such as coaches, teammates, family members, and professional medical teams (Owens et al., 2017). However, choking also has an internal psychological element (Mesagno and Beckmann, 2017), and any intervention to help athletes with choking problem to return to competitive sport should include both internal and external factors. Attribution training is believed to have the potential to help these athletes return to competitive sport as it is a process to change an athletes’ perception of failure and can be used to raise the confidence of athletes and help them have a positive mental attitude (Koivula et al., 2002). Therefore, in addition to external influences the study also takes into account individual characteristics, including personality traits and resilience levels, as well as well-established external factors such as environmental stressors, in designing the intervention. Consequently, this study sets out to accomplish four key goals: (1) to examine the effect of attribution training on the negative emotional responses of athletes who choke under pressure as they re-enter competitive environments; (2) to investigate the psychological determinants that influence athletes’ ability to return to competition after instances of choking; (3) to study the mediating roles of fear of failure and self-criticism based on choking process theory, and further explore the impact of attribution training on self-efficacy; (4) to propose suggestions for addressing the psychological issues of athletes experiencing choking.

Given the adverse effects of fear of failure and self-criticism on athletes, understanding their self-efficacy during sports is crucial. Compared to existing studies, this research’s contributions are threefold. First, it focuses on the self-efficacy of athletes experiencing the choking phenomenon. Second, the study seeks to analyze how the unique blend of physiological and psychological reactions to competition, specifically the fear of failure and self-criticism experienced by athletes who have previously choked, serves as intermediary factors influencing their self-efficacy. Third, drawing from the choking process theory, it’s noted that athletes’ perception of pressure (e.g., the demand to win titles or achieve certain rankings) triggers an interaction between fear of failure and self-criticism, amplifying the significance they place on competition outcomes. Consequently, this investigation employs fear of failure and self-criticism as intermediary variables and examines how attribution training, as a personal tool, can mitigate these feelings. It further explores how attribution training influences athletes’ belief in their capabilities, improving their ability to manage emotions and boost confidence, thereby offering both theoretical and practical insights for their re-engagement in competitive sports.

## 2 Literature review

### 2.1 Choking

Baumeister was the first social psychologist to simulate pressure in a laboratory setting to observe the phenomenon of choking (Baumeister, 1984). The process theory of choking posits that the occurrence of choking is a dynamic process from cognition to execution, depending on the comprehensive influence of multiple components (Christensen et al., 2015). During the cognitive phase, permanent attributes like self-perception and traits of anxiety, as well as fluctuating elements such as internal triggers and external stimuli, primarily influence the experienced stress levels (Carrigan and Barkus, 2016). The interaction between stress and self-perception and anxiety states amplifies the importance attributed to competition outcomes (Fisher and Zwart, 1982). Individuals with strong self-awareness and those with anxiety traits are more susceptible to the negative impact of external pressures, with athletes who fear losing or are overly concerned about audience reactions being more prone to choking (Wang et al., 2004).

Furthermore, the process theory of choking indicates that the phenomenon is a result of the combined action of self-perception, state anxiety, and coping methods, leading to the obstruction of automatic execution, preventing athletes from capturing competition-related information and reducing working memory (Beilock and Carr, 2001). Attribution training (positive feedback, increasing successful experiences, clarifying controllable factors, emphasizing personal effort, etc.) can improve training and competition results (Parker et al., 2016), reduce state anxiety (Han et al., 2022), and regulate negative self-awareness emotions (Flora et al., 2012). Individuals adopt certain methods, means, or ways to cope with external environmental demands and related distress (Folkman, 2008). Personal adoption of proactive coping methods and avoidance coping methods can reduce the phenomenon of choking (Diotaiuti et al., 2021). Especially, proactive coping methods motivate athletes to work harder, and correct attribution training can improve individuals’ resilience, enhance confidence, and encourage persistent learning (Parkes and Mallett, 2011). Gernigon and Delloye (2003) pinpointed the cause of failure to factors that are not only unstable but also manageable, including effort, strategy, practice, skill, or any variable element, facilitating athletes’ success in reaching their objectives post-failure. Therefore, it can be said that the fear of failure experienced by athletes undergoing the choking phenomenon may be adjusted through proper attribution training.

### 2.2 Attribution training, fear of failure, and self-criticism

Attribution training can reduce self-perception and anxiety by improving athletes’ stress cognition. Extensive research shows that participation in sports due to intrinsic reasons or motivations significantly impacts performance outcomes and behaviors (Cerasoli et al., 2014). Studies on intrinsic motivation, expectations, and attributions tell us that we should assist sports participants in setting challenging yet realistic goals, encourage them to emphasize effort and factors within their control during the goal achievement process. The result of employing these methods will foster a sense of competence and self-control in participants, generating a desire for continued success in sports (Tedesqui and Young, 2017). An analysis of the choking literature regarding the effects of stress manipulation suggests that while no studies have specifically compared the effectiveness of multiple sources of stress to a single source, Baumeister proposed that when multiple stress sources are applied simultaneously, their effects may be cumulative, indicating that the more numerous the sources of stress, the greater the overall perceived stress effect (Mesagno et al., 2019).

Attribution is the process of summarizing the causes and reasons leading to outcomes in order to understand the reasons behind developments or occurrences, thereby facilitating changes to these causes to ultimately improve outcomes. Turner et al. (2002) argue that students’ attributions for success and failure elicit a series of emotional reactions and changes in expectations. Attribution analysis is vital for learning because it entails an individual’s reflection on past behaviors and outcomes, leading to the summarization of lessons learned. This process significantly shapes subsequent actions.

Current research exploring self-criticism as a sensitivity factor to stress in relation to choking behavior is scarce. In sports competitions, especially at high levels, the effect of self-criticism needs attention. In sports psychology practice, strengthening interventions and assistance for self-criticism may help improve athletes’ performance (Ferguson et al., 2022). Sedikides and Luke (2008) proposed self-criticism as a self-defensive psychological tendency of individual performance is a personality factor for maintaining self-accuracy. Zuroff et al. (2016) considered self-criticism, as a personality phenomenon, to exhibit a psychological tendency toward sensitivity to negative information within habitual cognitive patterns. Research by Lueke and Skeel (2017) found that individuals with tendencies toward self-criticism, when faced with feedback on mistakes, would set higher standards for themselves to avoid potential magnification of errors. Thus, self-criticism, as an endogenous factor of the self-system, may influence behavioral performance in stress scenarios due to the introspection process.

In summary, attribution training, fear of failure, and self-criticism are theoretically highly related. From the perspective of the choking process theory, attribution training can reduce sources of stress for the individual. Therefore, attribution training, serving as a beneficial psychological tool, enables athletes to manage anxiety and fear of failure amidst competitive pressures effectively. It acts as a counterbalance to the adverse psychological states encountered by athletes who choke, diminishing their negative psychological responses as they persist in competition. Existing studies show that attribution training can effectively reduce anxiety, a major factor in fear of failure, suggesting a potential negative correlation between attribution training and fear of failure. Additionally, empirical findings reveal that negative emotions play a role in fostering self-criticism, but attribution training has been found to effectively modulate these negative emotions (Southall, 2016). This leads to the theory that attribution training may inversely relate to self-criticism. Based on these insights, this research introduces the following hypotheses for exploration:

*H1:* Attribution training is negatively correlated with fear of failure.

*H2:* Attribution training is negatively correlated with self-criticism.

### 2.3 Self-criticism and self-efficacy

Bandura (1977) first introduced the concept of self-efficacy, positioning it as a cornerstone in an individual’s belief system, crucial for stress and anxiety management. This self-belief, characterized by its ability to regulate emotions, modifies how one perceives and reacts to their goals and their achievability. It enables individuals to navigate their endeavors more effectively (Bandura et al., 2003). Self-efficacy is about having confidence in one’s emotional regulation capabilities, influencing emotional management success and laying the groundwork for emotional intelligence (Bandura et al., 2003). Current research is increasingly focused on boosting self-efficacy to reduce stress, foster emotional steadiness, control emotional responses, and support mental well-being (Paciello et al., 2016). It includes a sophisticated internal makeup that accounts for managing both negative and positive emotions (Caprara et al., 2008).

Lately, the link between stress and different types of psychopathology, such as anxiety and depression, has gained growing acknowledgment (Barlow, 2000). Stress represents the process of dynamic adjustment between disequilibrium and equilibrium when an individual’s internal homeostasis is threatened by stressors (Chrousos and Gold, 1992). Individuals prone to self-criticism, when faced with stress situations, due to introspection being activated, exhibit increased cognitive stress perception (Thompson and Zuroff, 2004). Cunha and Paiva (2012) observed the exam anxiety behaviors of 449 high school students, finding that relatively high levels of exam anxiety were significantly reflected in students with tendencies toward self-criticism. Powers et al. (2009) conducted pre- and post-season stress perception observations on 55 college athletes, examining the self-criticism trait as a correlate and found that athletes with tendencies toward self-criticism exhibited relatively high levels of cognitive worry and anxiety. Campos et al. (2018), through their observation of life event stress in 207 adults, discovered that subjects prone to self-criticism showed relatively high stress sensitivity. The emergence of stressors also signifies the activation of stress responses, aimed at restoring the body’s homeostasis, yet some athletes may experience significant negative emotions. Recent research has provided partial evidence for the mediating effect of regulatory self-efficacy on the relationship between negative emotions and behaviors (Mesurado et al., 2018). Thus, these studies also confirm a correlation exists between regulatory self-efficacy and self-criticism.

Fear of failure manifests as a distressing emotional response individuals encounter in pursuits of achievement, stemming from the foresight of failing to achieve set objectives (Caraway et al., 2003). At its heart, shame is identified as the fundamental element of the fear of failure (Conroy, 2004). The fear of failure is intricately linked with cognitive and self-perception elements, including low self-esteem and a deficiency in confidence, which are often paired with a potent motivation to avoid failure. Athletes frequently need to perform intricate motor tasks under social scrutiny and in demanding settings. The way athletes perceive their capacity to handle competitive stress contributes to the emergence of negative emotions, with fear of failure indicating potential disturbances in performance (Mellalieu et al., 2009), which in turn affects their performance levels. Many researchers have explored and established the link between attribution training and self-efficacy. It has been found that regulatory self-efficacy acts as a partial intermediary between attribution training and fear of failure (Relich et al., 1986). Moreover, when athletes experience success, it tends to elevate their goals and aspirations. However, if they experience failure, their self-efficacy will decline (Stoeber et al., 2008).

In summary, there’s a strong link between regulatory self-efficacy, fear of failure, and self-criticism, suggesting these factors are closely related in competitive scenarios. Choking theory posits that heightened personal stress perceptions fuel athletes’ fear of failure and self-criticism. A lower stress perception could reduce fear of failure, potentially increasing self-efficacy. Similarly, less self-criticism might boost regulatory self-efficacy. Hence, the study proposes related hypotheses based on these insights.

*H3*: Fear of failure is negatively correlated with self-efficacy.

*H4*: Self-criticism is negatively correlated with self-efficacy.

### 2.4 The mediating role of fear of failure and self-criticism

It appears that fear of failure influences how attribution training impacts self-criticism. Leary (2007) measured college students’ responses to emotional difficulties encountered in daily life, concluding that self-efficacy explained unique differences in predicting individuals’ adaptive emotional, thought, and response patterns to negative or emotionally challenging situations. Relative to athletic failure, self-efficacy can also play a crucial role in regulating emotional distress (Ceccarelli et al., 2019). When athletes self-criticize following their own failures, they may also incur physiological costs (Terry and Leary, 2011). The psychological and physical toll is significant for athletes who experience failure, leading to intense self-criticism, judgment, and rumination (Ceccarelli et al., 2019). Coreia et al. (2017) indicate that highly perfectionistic individuals, characterized by their unrealistically high expectations and self-criticism, are more susceptible to failure and exhibit more adverse cognitive, emotional, and performance reactions to failure compared to those with less perfectionistic tendencies. Highly self-critical individuals may overburden themselves in the process of achieving goals, thereby increasing the chance of failure (Hewitt and Flett, 1991).

Numerous researchers have explored and established the link between attribution training and self-efficacy. Stajkovic and Sommer (2000) highlighted that the sense of enhanced ability, a psychological state related to achievement, is influenced primarily by the locus of causality, which has a conceptual connection to self-efficacy. Bandura et al. (1999) argued that feedback on past performance can influence future self-efficacy and, consequently, future performance, based on whether the feedback is perceived as stemming from internal or external sources. Additionally, Stajkovic and Sommer (2000) discovered in their studies that a student’s previous academic achievements could predict their future success, influenced by their self-efficacy levels and whether they attribute their past achievements to internal or external factors.

Drawing from the principles of choking theory, should attribution training affect self-efficacy, then self-efficacy could serve as a mediator in its relationship with self-criticism. Gilbert et al. (2004) observed that individuals who are highly self-critical show a pronounced introspective awareness regarding their own goal fulfillment and frequently resort to self-correction or self-punishment. Research across various professional fields shows that regulatory self-efficacy not only impacts anxiety but also affects self-criticism. For example, self-efficacy helps women release and overcome self-critical traumatic experiences from past intimate partner violence experiences (Crapolicchio et al., 2021). Conroy et al. (2001) argue that athletes anticipate success because it increases others’ attention toward them. If they fail, others lose interest in them, meaning that something of value to themselves is taken away, thus constituting a threat and subsequently generating feelings of fear. Self-criticism has been extensively studied in emotional disorders, especially those characterized by depressive symptoms, although the literature remains limited. Self-criticism is thought to increase the risk of depression following perceived failures (Blatt and Zuroff, 1992). Self-critical individuals are more susceptible to core beliefs of worthlessness and inadequacy, feeling less valuable than others and inferior (Zuroff and Mongrain, 1987). Dunkley et al. (2003) established that self-critical perfectionism is associated with diminished self-efficacy.

Summarizing, within the framework of choking process theory, it’s identified that athletes’ competitive failures fuel choking by adversely affecting self-criticism. Evidence points to a strong link between fear of failure and attribution training, leading to the inquiry of whether attribution training influences self-efficacy through self-criticism. Further, it explores whether attribution training can alter emotional regulation self-efficacy by navigating through fear of failure and self-criticism. Thus, the study introduces two focused hypotheses for investigation:

*H5*: Fear of failure is negatively correlated with self-criticism.

*H6*: Fear of failure and self-criticism have a negative mediating effect on the relationship between attribution training and self-efficacy.

**Figure 1:**
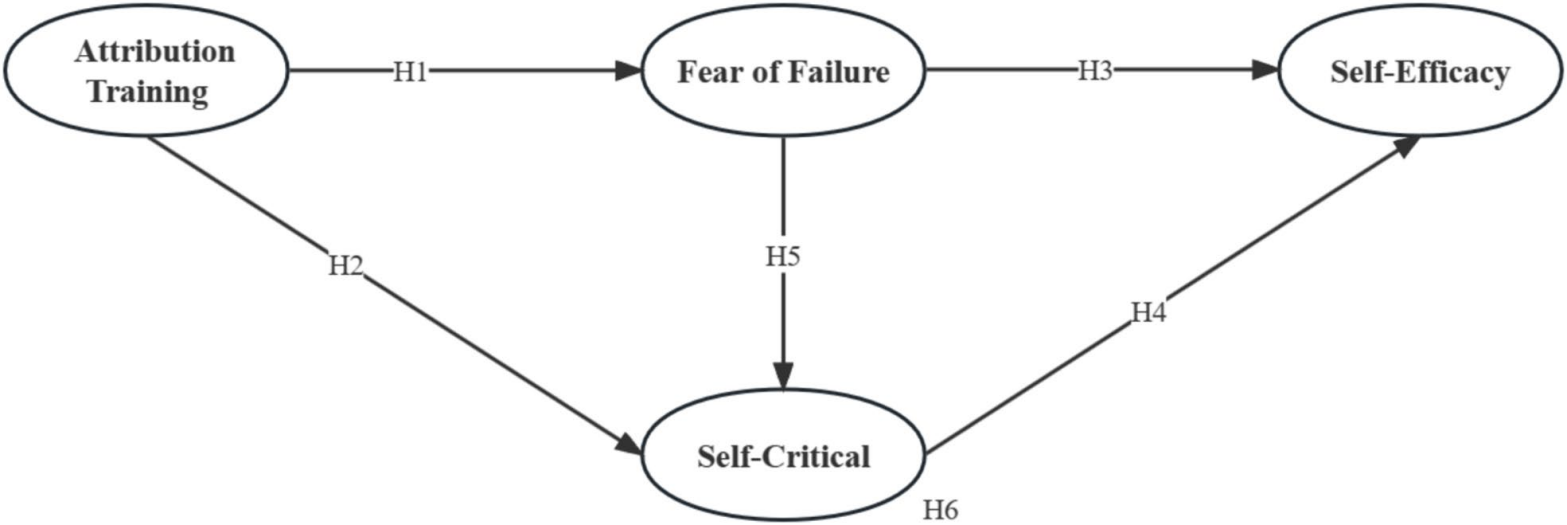
The proposed model.

All hypotheses are illustrated in Figure 1.

## 3 Methodology

### 3.1 Participants and procedure

The snowball sampling technique was used to collect data through the combination of online electronic questionnaires and offline paper questionnaires to explore the choking phenomenon among elite athletes (defined as those at the national second level or higher). Participants all self-reported themselves as choking-susceptible athletes due to various reasons. Before the survey, the ethics committee at the first author’s institution approved the research protocol. The research team conducted a survey of elite athletes in Central China between October and December 2023. In this study, 350 questionnaires were distributed, 350 questionnaires were collected after the questionnaires were distributed, and 328 valid questionnaires were finally eliminated through screening. And the relevant statistical analysis is carried out on the data.

The demographic breakdown of the participants, as detailed in Table 1, shows that nearly 70% were between 18 and 23 years old; males constituted 61.9%, and females 38.1%; 77.4% were classified as tier 2 athletes, with a majority (62.8%) participating in ball sports.

**Table 1:** Athlete participant demographics (*N* = 328).

Profiles	Survey (%)
Age	
<18	(65) 19.8%
18–22	(210) 64.0%
>22	(53) 16.2%
Gender	
Male	193 (59%)
Female	135 (41%)
Education level	
High school diploma	80 (24.4%)
Bachelor’s degree	178 (54.3%)
Graduate degree	70 (21.3%)
Sport level	
Tier 1 athlete	27 (8.2%)
Tier 2 athlete	216 (65.9%)
Tier 3 athlete	85 (25.9%)
Sports items	
Ball sports	153 (46.6%)
Athletics events	101 (30.8%)
Water sports	18 (5.5%)
Other sports	56 (17.1%)

### 3.2 Measures

The questionnaire used in this study is rigorously structured, consisting of five separate and interrelated sections covering a total of 24 specific items. The first part focuses on collecting the basic demographic information of the participants, including age, gender, education level and sports level, etc., to ensure the diversity and representation of the sample. The second part draws on the self-criticism scale compiled by Thompson and Zuroff (2004), and selects five items to evaluate the participants’ self-cognition tendency. Example entry: “You believe that others will treat you with the respect you deserve, even when they do not know you well.” The design aims to delve into the participants’ inner psychological states. The third part focuses on attribution training, collecting data by using six key items in the attribution Training Scale developed by Millar and Shevlin (2007). For example: “Do you think that chance and luck play a large role in determining whether you achieve your goals?” None of our participants had received attribution training before they were tested. This session aims to understand the participants’ cognitive patterns regarding the causes of success and failure. The fourth part focuses on athletes’ self-efficacy, measured using six items from a scale carefully designed by Chesney et al. (2006). A good example: “Even in the face of adverse circumstances, you maintain a positive attitude and believe that you can overcome the difficulties.” This section aims to assess an athlete’s mental resilience and self-motivation in the face of challenges. Finally, the fifth part uses the five items of Conroy (2003) original self-criticism scale to analyze the fear of failure of athletes. Example entry: “When you fail, do you worry excessively about what others think of you?” This link is of great significance for understanding the psychological reaction of athletes under competitive pressure. All four scales were scored strictly on a five-point Likert scale, ranging from 1 (“strongly disagree, never “) to 5 (“strongly agree “) to ensure data accuracy and comparability.

Modifications were made to these scales to better fit the specific cultural context and the field of study. By searching the Chinese journal database, China Journal Network, Web of Science and other literature materials, this paper provides a strong theoretical basis for the experimental research. Expert interview method is used to consult the feasibility and rationality of the implementation of attribution training for choking athletes, as well as the modification of the questionnaire and other issues that need attention, and then verify the reliability of the revised scale. In order to ensure the reliability of the revised scale, the research team conducted a pilot test on elite college athletes from three universities in Changsha city with convenient sampling method (Fornell and Larcker, 1981). Of the 80 questionnaires distributed, 75 were successfully recovered and confirmed to be valid, and the effective recovery rate reached a high level. In addition, the Cronbach *α* coefficient test results of all scales show that the αcoefficient of all scales is stable above 0.9, which fully proves that the research team’s modification of the scale is reasonable and effective.

### 3.3 Data analysis

The study used Structural Equation Modeling (SEM) with Maximum Likelihood Estimation via AMOS version 26.0 for model analysis. SEM, a preferred technique for assessing hidden variables and testing relationship theories among these variables, was applied in a two-step approach as suggested by Anderson and Gerbing (1988). This involved first evaluating the overall validity of both the measurement and structural models using SEM. After establishing validity, the study then examined the fit indices and path coefficients to confirm the hypothesized model’s predictions.

## 4 Results

### 4.1 Measurement model

According to Fornell and Larcker (1981), reliability assessments should include Cronbach’s alpha and Composite Reliability (CR) to evaluate latent variables. Table 2 shows Cronbach’s alpha values ranging from 0.830 to 0.890 and CR values from 0.830 to 0.892, both exceeding the recommended threshold of 0.7, indicating strong reliability.

**Table 2:** Assessment of reliability and validity.

Items	Loadings	Cα	AVE	CR
Attribution training (AT)		0.830	0.553	0.830
AT1	0.831			
AT2	0.828			
AT3	0.638			
AT4	0.655			
Fear of failure (FF)		0.842	0.575	0.844
FF1	0.778			
FF2	0.766			
FF3	0.803			
FF4	0.682			
Self-criticism (SC)		0.890	0.623	0.892
SC1	0.775			
SC2	0.764			
SC3	0.808			
SC5	0.817			
SC6	0.780			
Self-efficacy (SE)		0.877	0.591	0.878
SE1	0.718			
SE2	0.749			
SE3	0.784			
SE4	0.826			
SE5	0.763			

For convergent validity, which assesses if different methods measuring the same characteristic align, factor loadings and Average Variance Extracted (AVE) were used. Results in Table 3 show factor loadings between 0.830 and 0.890 and AVE values from 0.553 to 0.623, above the advised 0.5, ensuring high convergent validity.

**Table 3:** Evaluation of discriminant validity.

Construct	AT	FF	SC	SE
AT	0.744			
FF	−0.466**	0.759		
SC	−0.557**	0.450**	0.789	
SE	0.459**	−0.384**	−0.458**	0.769

The diagonal shows the square root of the AVE, while off-diagonal elements represent Pearson’s correlations between constructs. ** *p* < 0.01.

Discriminant validity, checking if constructs differ, was confirmed as all correlation coefficients are below the square root of the AVE for each variable, demonstrating adequate discriminant validity.

### 4.2 Common method variance

The study addressed the concern of Common Method Variance (CMV) in behavioral research effectively. Utilizing Harman’s single-factor test, it found that only 40.798% of the variance was extracted, which is under the 50% threshold, suggesting the absence of CMV (Podsakoff et al., 2012). Secondly, by applying Lindell and Whitney (2001) comparison method between single-factor and multi-factor models, a significant chi-square value difference was observed (1287.6 for single-factor with 135 degrees of freedom versus 286.3 for multi-factor with 129 degrees of freedom), with a ratio of 166.9. This substantial discrepancy highlighted the difference between the models, further indicating no CMV presence, thus negating the need for CMV correction.

### 4.3 Structural path model

The absence of negative values in error terms and residuals within the structural model indicates compliance with basic fit test standards. As per Hair et al. (2010), the structural model demonstrated favorable fit indices (*χ*^2^/df = 2.289, GFI = 0.907, NFI = 0.909, CFI = 0.946, TLI = 0.937, RMSEA = 0.063). Table 3 displays significant correlations among independent, mediating, and dependent variables, supporting the research hypotheses.

**Figure 2:**
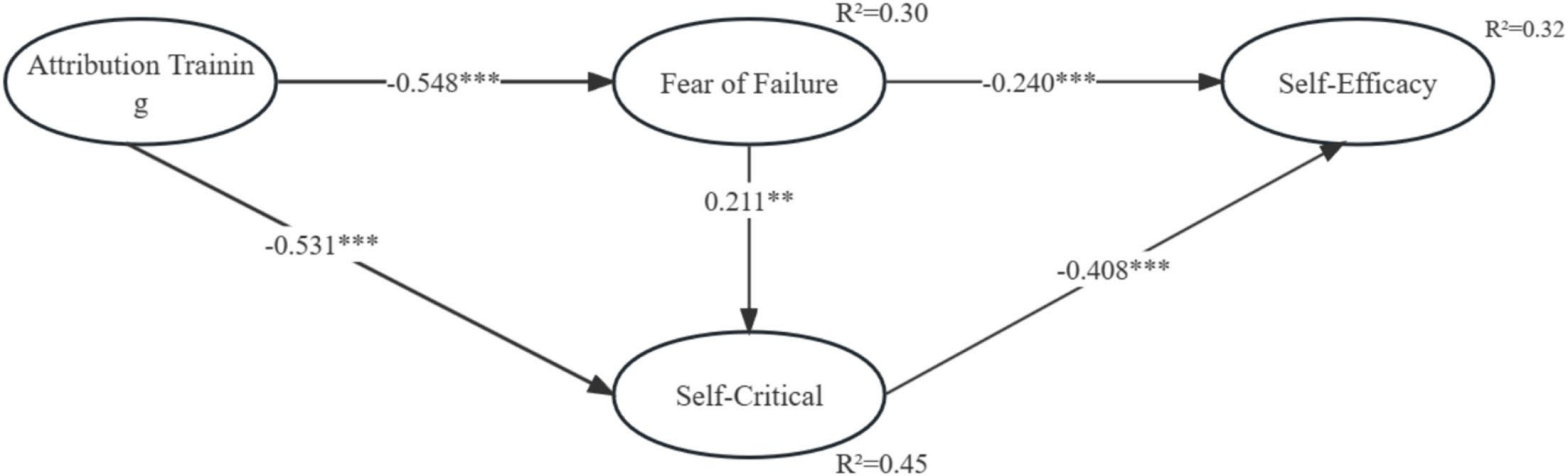
Path model analysis. *** *p* < 0.001.

Results from the structural path model (Figure 2) reveal significant impacts of attribution training on fear of failure (*β* = −0.548, *p* < 0.001) and self-criticism (*β* = −0.531, *p* < 0.001), validating H1 and H2. Fear of failure significantly influences self-efficacy (*β* = −0.240, *p* < 0.001, H3), as does self-criticism (*β* = −0.408, *p* < 0.001, H4). Additionally, fear of failure significantly impacts self-criticism (*β* = −0.211, *p* < 0.01, H5).

The conceptual model suggests that attribution training positively influences athletes’ self-efficacy via mediating factors like fear of failure and self-criticism. Employing the bootstrapping method recommended by Bollen and Stine, the study validated the mediation effect. Table 4 presents a significant mediating effect of fear of failure and self-criticism between attribution training and self-efficacy (standardized indirect effect = 0.395, *p* < 0.001, H6). These findings suggest that athletes undergoing attribution training experience notable reductions in fear of failure and self-criticism, resulting in improved self-efficacy during competition, along with enhanced positive emotions and reduced negative emotions.

**Table 4:** Mediation analysis (5,000 bootstrap samples).

	Point estimate	Product of coefficients		Bootstrapping		
				Bias-corrected 95% CI		Two-tailed significance
		SE	*Z*	Lower	Upper	
AT→FF → SC → SE	0.395	0.047	8.404	0.306	0.490	< 0.001

## 5 Discussion

### 5.1 Theoretical contribution

This study explores how attribution training interventions impact athletes’ psychological factors: fear of failure, self-criticism, and self-efficacy. The findings reveal a significant decrease in fear of failure with attribution training. Additionally, fear of failure and self-criticism mediate the relationship between attribution training and self-efficacy. Athletes undergoing attribution training experience notable reductions in fear of failure and self-criticism, resulting in improved self-efficacy during competition, along with enhanced positive emotions and reduced negative emotions. The influence of attribution training was most significant on fear of failure, followed by self-criticism. As illustrated in Figure 2, the extended choking model accounted for 32% of the variance in self-efficacy. These findings align with prior studies suggesting that attribution training can mitigate fear of failure and self-criticism among athletes, particularly those encountering stress-induced choking incidents (Hill and Shaw, 2013). Additionally, attribution training interventions contribute positively to athletes’ psychological welfare and emotional regulation when facing choking situations. This is consistent with DM Hill ‘s findings, with a lack of perceived control/learned helplessness appearing to encourage multiple and chronic choking episodes, it would also be advantageous for practitioners to introduce attribution retraining to those athletes vulnerable to choking (Hill et al., 2019). Mento et al. (2023) study came to similar conclusions, the detection of learning motivation in relation to the choking effect as an important motivational construct in learning outcomes, being related to personality variables, emotional intelligence, and attribution training. The findings suggest that athletes’ psychological issues can be alleviated through means other than counseling, with attribution training being a more effective method.

This study contributes theoretically to the understanding of attribution training for athletes encountering choking incidents, reinforcing its role in bolstering self-efficacy within the framework of choking process theory. Firstly, these results supplement (Taggar and Neubert, 2004) assertion regarding the pivotal role of attribution training in optimizing cognitive capacities. In recent years, a large number of scholars have increasingly realized that mental exercise plays an important role in the learning and improvement of sports skills. Most relevant studies have achieved important results in the application of psychological techniques such as mental image, self-efficacy, goal setting and attention strategy to sports, and highlighted significant effects in various sports events. Orbach et al. (1999) also proposed the use of attribution training to enhance the athletes’ anti-pressure ability. The use of real-time feedback mechanism to help the human body to enhance the correlation of physical and mental cognition, and effectively maintain the dynamic balance of the optimal activation of the target brain wave, so as to prevent the choking effect and improve sports performance (Mesagno et al., 2008). They also resonate with the findings of Markman et al. (2006), who applied choking process theory to explicate attribution training as a specific cognitive mechanism enhancing positive psychological resources in professional settings. Similar results were found by Diotaiuti et al. (2021), who investigated choking episodes in archery and emphasized the significance of psychological interventions in mitigating performance declines. Secondly, this study advances existing models by employing choking process theory to elucidate mediating factors, providing clearer insights into how attribution training enhances self-efficacy for athletes facing choking episodes.

Theoretically, these findings offer additional evidence to choking theory, which suggests that the role of attribution training seems particularly important among athletes experiencing choking phenomena characterized by fear of failure and self-criticism. Furthermore, the theoretical significance of this study resides in its examination of the influence of attribution training on self-efficacy. This view is consistent with the results of many studies, such as Gernigon and Delloye (2003) found that attributing low performance to personal limitations hurts their self-efficacy. After studying college students’ achievement goals, attribution styles and academic self-efficacy, Turbeville-McCorry (2023) concluded that behavioral self-efficacy is significantly negatively correlated with failure avoidance attribution. The findings showed teacher perceptions and self-efficacy may be influenced by the attribution for the student’s behavior. The results suggest that avoidant and inhibited young people attribute social success and failure to stable internal causes and also have lower degrees of self-efficacy for social interactions (Innes and Thomas, 1989). Salanova et al. (2012) also concluded that internal attribution has an indirect positive predictive effect on learning strategies through self-efficacy, while external attribution can either have an indirect negative effect on learning strategies through self-efficacy or directly predict the level of learning strategies. There is a significant regression effect between behavioral self-efficacy, achievement goal and failure avoidance attribution, that is, there is a mutual causal relationship (Kogut, 2016). Within the framework of choking theory, fear of failure and self-criticism are regarded as sport-related traits contributing to the heightened stress perceived by athletes when resuming competition following choking incidents. Attribution training, functioning as a cognitive tool, appears effective in mitigating competitive stress for athletes resuming competition post-choking incidents. This sheds light on the individual traits of athletes experiencing choking episodes and underscores the significance of attribution training in managing stress within competitive sports. The findings not only enrich our understanding of attribution training’s role in addressing the challenges faced by athletes prone to negative emotions but also fill a gap in empirical evidence. This study extends the scope of existing research by Gray (2020), suggesting that specific circumstances influencing attribution training may vary in their impact on enhancing self-efficacy among athletes recovering from choking incidents.

### 5.2 Practical implications

This study advocates implementing intervention measures to assist athletes grappling with choking incidents in mitigating competition stress, a concern particularly pertinent within China’s competitive sports industry. Recognizing that athletes’ setbacks in training and competition invariably trigger negative emotions, several recommendations emerge from the study’s findings.

Firstly, national sports administrations are urged to bolster policy implementation, refine training methodologies, and optimize the efficacy of attribution training, given its positive impact on fear of failure, self-criticism, and self-efficacy. In countries experiencing rapid psychological development, there exists a heightened prevalence and accuracy of attribution training. Engaging in academic exchanges with such nations could foster improvements in the professionalism and standards of attribution training. Additionally, fostering collaborations between high-level sports teams and academic institutions could facilitate the establishment of comprehensive psychological training systems for athletes, encompassing attribution training. This would enable athletes to adeptly regulate their mental states and seamlessly engage in future training and competitions.

Secondly, national sports administrations ought to augment the allocation of funds for attribution training within daily training regimens. This necessitates an initial expansion in the procurement of mental and sports assessment equipment. Learning attribution training techniques requires equipment for stable, precise long-term data to tailor training plans and monitor athletes’ progress. By focusing on adjustable factors like skill improvement, strength, flexibility, and recovery, athletes’ self-efficacy can be boosted. Sports organizations should increase attribution training resources, support mental health advancement, and heighten awareness of its importance for optimal performance.

Thirdly, coaches play a pivotal role in enhancing athletes’ attribution training through their grasp of psychological principles. Thus, sports administrations need to embed further psychological training into coaching curriculums to advance attribution training skills. By prioritizing coaches, we can elevate athletes’ comprehension levels and optimize training efficacy in attribution techniques. This approach facilitates obtaining timely feedback on the nexus between athletes’ individual athletic and psychological states, fostering improvements in training methodologies, and mitigating athletes’ fear of failure and self-criticism. Consequently, this cultivates a heightened ability to regulate emotions and sustain self-efficacy levels.

Fourthly, it holds considerable practical significance to offer attribution training interventions to athletes, aiming to alleviate the detrimental impacts of choking phenomena on their emotions and mental well-being. Given the study’s revelations affirming the favorable correlation between attribution training and self-efficacy, coaches ought to mentor athletes grappling with choking phenomena in attribution training, aiding them in refining and honing their attribution skills. Athletes should be encouraged to engage in reflection and attribution analysis during periods of rest or prior to training and competitions, facilitating relaxation amidst the pressures of athletic endeavors. Thus, attribution training emerges as a viable intervention strategy for athletes contending with choking phenomena, fostering an enhancement in their self-efficacy as they prepare to reenter competitive arenas.

### 5.3 Limitations

The study first faced limitations due to its reliance on non-random sampling, which, although time-efficient, might have introduced bias into the sample’s representativeness. To mitigate this, future efforts should implement random sampling to ensure a more equitable distribution across the population, thereby improving sample representativeness and accuracy. Additionally, the study’s approach to attribution training was somewhat basic. Subsequent research should delve deeper into attribution training’s facets, such as its potential for stress reduction, cognitive therapy enhancements, and applications in dialectical behavior therapy. Moreover, the absence of alternative models in the current study opens avenues for future research to expand on these findings and explore more varied hypotheses. Finally, to broaden the applicability of its outcomes, it would be beneficial for future studies to consider enlarging the sample size.

## 6 Conclusion

This research underscores the significance of attribution training in helping high-level athletes manage emotions and improve performance by addressing fear of failure and self-criticism after choking phenomena. It demonstrates that such training not only fosters a positive sports attitude but also strengthens self-efficacy, confidence, and willpower, directly influencing athletes’ ability to overcome psychological challenges. The findings advocate for incorporating attribution training into athletes’ routines to enhance mental well-being, emotional control, and competitive readiness. Coaches and athletes are encouraged to focus on mental health as much as physical training, highlighting the crucial role of psychological resilience in sports success.

## Data Availability

The raw data supporting the conclusions of this article will be made available by the authors, without undue reservation.
